# What is the job satisfaction and active participation of medical staff in public hospital reform: a study in Hubei province of China

**DOI:** 10.1186/s12960-015-0026-2

**Published:** 2015-05-16

**Authors:** Pengqian Fang, Zhenni Luo, Zi Fang

**Affiliations:** School of Health and Medicine Management, Tongji Medical College, Huazhong University of Science and Technology, 13 Hangkong Road, Qiaokou District, Wuhan, 430030 China; School of Health Management, Guangzhou Medical University, 195 Dongfeng West Road, Yuexiu District, Guangzhou, 510182 China; The London School of Economics and Political Science, 13420, Houghton Street, London, WC2A 2AE UK

**Keywords:** Medical staff, China, Public hospital reform, Working situation, Satisfaction, Understanding, Perception

## Abstract

**Background:**

In China, public hospital reform has been underway for almost 5 years, and 311 pilot county hospitals are the current focus. This study aimed to assess the job satisfaction and active participation of medical staff in the reform. A total of 2268 medical staff members in pilot and non-pilot county hospitals in Hubei, China, were surveyed.

**Methods:**

Questionnaires were used to collect data. The Pearson chi-square statistical method was used to assess the differences between pilot and non-pilot county hospitals and identify the factors related to job satisfaction as well as the understanding and perception of the reform. Binary logistic regression was performed to determine the significant factors that influence the job satisfaction of medical staff in pilot county hospitals.

**Results:**

Medical staff members in pilot county hospitals expressed higher satisfaction on current working situation, performance appraisal system, concern showed by leaders, hospital management, and compensation packages (*P* < 0.05). They were exposed to work-related stress at a higher extent (*P* < 0.05) and half of them worked overtime. Within pilot county hospitals, less than half of the medical staff members were satisfied with current job and they have evidently less satisfaction on compensation packages and learning and training opportunities. The working hours and work stress were negatively related to the job satisfaction (*P* < 0.05). Satisfaction on the performance appraisal system, hospital management, compensation packages, and learning and training opportunities were positively related to job satisfaction (*P* < 0.05). Medical staff in pilot county hospitals exhibited better understanding of and more positive attitude towards the reform (*P* < 0.05).

**Conclusions:**

Pilot county hospitals have implemented some measures through the reform, but there still are deficiencies. The government officials and hospital administrators should pay attention to influencing factors of job satisfaction and focus on the reasonable demands of medical staff. In addition, the medical staff in pilot county hospitals exhibited a better understanding of the public hospital reform programme and showed more firm confidence, but there still were some medical staff members who hold negative attitude. The publicity and education of the public hospital reform still need improvement.

## Background

In October 2008, a draft of Healthcare Reform Plan was published by the Chinese government, and comments were solicited from the whole society. In this way, the general public was able to participate in policy-making in China, and this initiative is unprecedented [1,2]. In March 2009, China’s Healthcare Reform Plan was formally released to reduce the residents’ economic burden for medical services, mitigate the difficulties in affordability and accessibility of medical service, and provide safe, effective, convenient, inexpensive health services for universal coverage. The short-term goals of the health-care reform comprise five key programmes: expedite the establishment of a basic medical security system, initiate a national basic drugs system, improve the primary health-care service system, promote the gradual equalization of basic public health services, and implement a pilot programme of public hospital reform.

The pilot reform of public hospitals includes the following contents: improve health service systems and establish medical cooperative activities between urban and rural, substantially increase financial investment into hospitals, remove the provision of commercial hospital services, reform the compensation system of public hospitals, restructure the management systems of public hospitals, enhance income distribution and incentive programmes, and encourage social organizations to open hospitals [3]. Former premier Wen Jiabao considered public hospital reform as one of the most important and challenging tasks in the new health reform. Public hospitals are the principal medical and health service institutions in China. As such, these institutions are essential for public welfare in medical and health services. Therefore, public hospital reform is an important factor for the realization of health-care reform in China.

The pilot programme of county hospital reform is the threshold and emphasis of public hospital reform, and county hospitals are currently in focus. China’s Ministry of Health selected 311 county hospitals on a national scale as pilot hospitals, and 20 of the 311 are located in Hubei province. These 311 pilot county hospitals must take the lead to carry out the measures and contents of public hospitals reform mentioned above, while the rest of the county hospitals could remain original.

County hospitals are considered as flagships of three-tiered rural health-care systems and the main provider of health services; these services include treatment and emergency services, disease prevention, vaccination, health education, maternal and child health-care services, and reproductive services for rural residents [4], who account for more than 50% of the total Chinese population, and some urban residents. In China, a county hospital is generally equipped with 300 to 500 beds and approximately 400 to 600 medical staff members, providing health services for approximately 0.5 million individuals in an area of 2000 to 4000 km^2^.

Medical staff is the direct provider of hospital services and key element in the development of health services. The work performance, attitude, and commitment of medical staff directly influence the outcomes of health service delivery, such as medical safety, service quality, doctor–patient relationship, patient satisfaction, and hospital management, particularly the operating efficiency and effectiveness of a hospital. Medical staff members in pilot county hospitals are also responsible for the implementation and promotion of the reform policies as well as the generation of support for the reform.

To our knowledge, studies on the working situation, satisfaction, and attitude towards the reform of medical staff in pilot county hospitals in China in this specific period are few [5,6], let alone on comparison between pilot and non-pilot county hospitals. The purposes of this study are to describe and compare the working situation, satisfaction, and attitude towards the reform of medical staff in pilot county hospitals with those of medical staff in non-pilot county hospital in Hubei province and to analyse and determine the influencing factors of medical staff in pilot county hospitals. We aim to improve job satisfaction and arouse active participation in public hospital reform, to provide high-quality medical services to residents.

## Methods

### Study population

County hospitals were selected by sampling at several stages. Considering that Hubei province is a large area in central China, we initially selected seven cities in east, south, west, north, northwest, northeast, and central Hubei according to geographical locations and economic development levels. They are Wuhan city, Jingzhou city, Yichang city, Xiangyang city, Shiyan city, Suizhou city, and Jingmen city, respectively. There are 20 pilot county hospitals in Hubei province. Two pilot counties and one non-pilot county were then randomly selected from each of the seven cities. One county hospital was finally chosen from each of the 21 counties. Thus, this study comprised 21 county hospitals, including 14 pilot county hospitals and 7 non-pilot county hospitals.

In each selected county hospital, 110 medical staff members, including doctors, nurses, and medical technicians (personnel in pharmacy, clinical laboratory, and radiology department) were randomly chosen and considered as subjects of the study. A total of 2310 medical staff members from 1531 in pilot county hospitals and 737 in non-pilot county hospitals were investigated and 2268 valid answer sheets were returned, resulting in a response rate of 98.18%. This study was approved by the Ethics Committee of Tongji Medical College, Huazhong University of Science and Technology (IRB No: FWA00007304). The medical staff was aware of this study and willing to participate. The privacy of the investigated medical staff was strictly protected by filling in the questionnaires anonymously.

### Questionnaire

Adapting the Minnesota Satisfaction Questionnaire based on the actual situation of medical staff members of county hospitals in China, this study formulated a questionnaire and its index system through questionnaire investigation, literature survey, and Delphi expert consultation. The questionnaire consisted of three parts: Part 1, sociodemographic information; Part 2, working situation and satisfaction; and Part 3, understanding and perception of the reform. Through reliability statistics, Cronbach’s alpha is 0.913.

Part 1 included sociodemographic information of gender, age, educational background, position, professional title, and years in professional working experience.

In Part 2, working situation included two aspects: the number of hours spent at work every day and the work stress one felt. For the number of hours, six options were provided from “≤8 h” to “≥12 h”. For work stress, five options were provided from “no pressure” to “extreme pressure”. In Part 2, satisfaction included six aspects: current job, performance appraisal system, concern showed by leaders, hospital management, compensation packages, and learning and training opportunities. For these aspects, five options (numbered from 1 to 5) were provided to express satisfaction degree: 1, very satisfied; 2, satisfied; 3, moderate/acceptable; 4, dissatisfied; and 5, very dissatisfied.

In Part 3, investigation on the understanding and perception of the reform included the following: understanding of the specific contents of the public hospital reform programme, perceived level of reform promotion, changes experienced before and after the reform introduction in 2008, and the perception of the effect of the public hospital reform.

### Statistical analysis

EpiData3.1 was used to establish a database, and double machine inputting method was used to enter the collected data into the computer. PASW Statistics 18.0 was used to perform statistical data analysis. The sociodemographic factors of the investigated medical staff were summarized using a descriptive statistical analysis method. The Pearson chi-square statistical method was used to assess the differences between medical staff in pilot county hospitals and medical staff in non-pilot county hospitals in working situation, satisfaction on job and wok-related factors, and the understanding and perception of the reform. The Pearson chi-square statistical method was also used to analyse the factors influencing job satisfaction and the understanding and perception of the reform in pilot county hospitals. Binary logistic regression was then performed to determine significant factors influencing job satisfaction in pilot county hospitals. The dependent variable (the outcome of interest) in logistic regression included the following: 1 “satisfied” (very satisfied, satisfied, moderate) and 0 “otherwise” (dissatisfied, very dissatisfied). We substituted the variables with statistical significance determined from the Pearson chi-square statistical test into the binary logistic regression model for calculation (*P* < 0.05). Odds ratio (OR) was reported at a 95% confidence interval (CI) where appropriate. All of the tests were conducted at 5% significance level.

## Results

### Sociodemographic characteristics of the investigated medical staff in pilot and non-pilot county hospitals

The investigated medical staff members were mainly comprised of females (66.58%). The largest proportion of age of the medical staff ranged from 25 to 34 (38.27%). The largest proportion of educational background was bachelor’s degree (48.19%), and the largest proportion of professional title was middle title (43.83%; Table 1). In this study, different professional titles showed various professional skill levels of the medical staff members. In China, a medical staff member needs to undergo and pass a special qualification examination and assessment to achieve his (or her) professional title corresponding to a particular professional skill level.

**Table 1:** **Sociodemographic characteristics of medical staff in pilot and non-pilot county hospitals**

**Characteristic**	**Pilot county hospitals**		**Non-pilot county hospitals**		**Total**	
	**Medical staff (** ***n*** **= 1531)**	**Percentage (%)**	**Medical staff (** ***n*** **= 737)**	**Percentage (%)**	***n***	**%**
Gender						
Male	552	36.05	206	27.95	758	33.42
Female	979	63.95	531	72.05	1510	66.58
Age						
24 and below	150	9.80	63	8.55	213	9.39
25–34	527	34.42	341	46.27	868	38.27
35–44	559	36.51	212	28.77	771	33.99
45–54	268	17.50	107	14.52	375	16.53
55 and above	27	1.76	14	1.90	41	1.81
Educational background						
Technical secondary school and below	188	12.28	48	6.51	236	10.41
Junior college	547	35.73	216	29.31	763	33.64
Bachelor’s degree	737	48.14	356	48.30	1093	48.19
Master’s degree or above	59	3.85	117	15.88	176	7.76
Position						
Doctor	599	39.12	280	37.99	879	38.76
Nurse	661	43.17	357	48.44	1018	44.89
Medical technician	271	17.70	100	13.57	371	16.36
Professional title						
No title	24	1.57	11	1.49	35	1.54
Junior title	513	33.51	333	45.18	846	37.30
Middle title	729	47.62	265	35.96	994	43.83
Senior title	265	17.31	128	17.37	393	17.33
Years in professional working experience						
1–5 years	288	18.81	196	26.59	484	21.34
6–10 years	214	13.98	141	19.13	355	15.65
11–15 years	227	14.83	81	10.99	308	13.58
16–20 years	307	20.05	135	18.32	442	19.49
20 years and above	495	32.33	184	24.97	679	29.94
City						
Wuhan city	220	14.37	108	14.65	328	14.46
Jingzhou city	218	14.24	105	14.25	323	14.24
Yichang city	215	14.04	98	13.30	313	13.80
Xiangyang city	220	14.37	104	14.11	324	14.29
Shiyan city	219	14.30	108	14.65	327	14.42
Suizhou city	219	14.30	105	14.25	324	14.29
Jingmen city	220	14.37	109	14.79	329	14.51

### Working situation, job satisfaction, and satisfaction on work-related factors of medical staff in pilot and non-pilot county hospitals

In pilot county hospitals, 51.86% of the medical staff members work for more than 8 h a day and 55.39% are exposed to considerable pressure or extreme pressure. The percentages of medical staff members in pilot county hospitals dissatisfied or very dissatisfied with current job, performance appraisal system, concern showed by leaders, hospital management, compensation packages, and learning and training opportunities were 17.37%, 15.55%, 10.65%, 6.53%, 35.41%, and 47.42%, respectively.

Through Pearson chi-square statistical analysis, the results showed that medical staff in pilot and non-pilot county hospitals exhibited significant differences in work stress and satisfaction on the following aspects: current job, performance appraisal system, concern showed by leaders, hospital management, and compensation packages (*P* < 0.001; Table 2).

**Table 2:** **Working situation, job satisfaction, and satisfaction on work-related factors of medical staff in pilot and non-pilot county hospitals**

**Working situation and satisfaction**	**Pilot county hospitals (** ***n*** **= 1531)**		**Non-pilot county hospitals (** ***n*** **= 737)**		**Total**		***χ*** ^**2**^	***P***
	***n***	**%**	***n***	**%**	***n***	**%**		
The number of hours spent at work everyday								
8 h and below	737	48.14	394	53.46	1131	49.87		
9 h	407	26.58	171	23.20	578	25.49		
10 h	219	14.30	103	13.98	322	14.20		
11 h	46	3.00	14	1.90	60	2.65		
12 h	37	2.42	16	2.17	53	2.34		
12 h and above	85	5.55	39	5.29	124	5.47	7.582	0.181
The degree of work stress								
No pressure	31	2.02	168	22.80	199	8.77		
Slight pressure	411	26.85	157	21.30	568	25.04		
Moderate pressure	241	15.74	150	20.35	391	17.24		
Considerable pressure	682	44.55	198	26.87	880	38.80		
Extreme pressure	166	10.84	64	8.68	230	10.14	299.217	<0.001
Satisfaction on current job								
Very satisfied	136	8.88	38	5.16	174	7.67		
Satisfied	564	36.84	205	27.82	769	33.91		
Moderate	565	36.90	313	42.47	878	38.71		
Dissatisfied	239	15.61	158	21.44	397	17.50		
Very dissatisfied	27	1.76	23	3.12	50	2.20	38.744	<0.001
Satisfaction on performance appraisal system								
Very satisfied	34	2.22	12	1.63	46	2.03		
Satisfied	475	31.03	101	13.70	576	25.40		
Moderate	784	51.21	404	54.82	1188	52.38		
Dissatisfied	197	12.87	180	24.42	377	16.62		
Very dissatisfied	41	2.68	40	5.43	81	3.57	111.369	<0.001
Satisfaction on concern showed by leaders								
Very satisfied	76	4.96	11	1.49	87	3.84		
Satisfied	517	33.77	134	18.18	651	28.70		
Moderate	775	50.62	433	58.75	1208	53.26		
Dissatisfied	133	8.69	117	15.88	250	11.02		
Very dissatisfied	30	1.96	42	5.70	72	3.17	109.148	<0.001
Satisfaction on hospital management								
Very satisfied	77	5.03	13	1.76	90	3.97		
Satisfied	608	39.71	162	21.98	770	33.95		
Moderate	746	48.73	417	56.58	1163	51.28		
Dissatisfied	83	5.42	118	16.01	201	8.86		
Very dissatisfied	17	1.11	27	3.66	44	1.94	145.094	<0.001
Satisfaction on compensation packages								
Very satisfied	20	1.31	8	1.09	28	1.23		
Satisfied	264	17.24	40	5.43	304	13.40		
Moderate	705	46.05	227	30.80	932	41.09		
Dissatisfied	460	30.05	336	45.59	796	35.10		
Very dissatisfied	82	5.36	126	17.10	208	9.17	189.192	<0.001
Satisfaction on learning and training opportunities								
Very satisfied	34	2.22	11	1.49	45	1.98		
Satisfied	246	16.07	101	13.70	347	15.30		
Moderate	525	34.29	252	34.19	777	34.26		
Dissatisfied	481	31.42	247	33.51	728	32.10		
Very dissatisfied	245	16.00	126	17.10	371	16.36	4.193	0.38

### Analysis and determining of the significant factors influencing the job satisfaction of medical staff in pilot county hospitals

In pilot county hospitals, only 45.72% of medical staff expressed that they were very satisfied or satisfied with current job. About 36.90% of medical staff felt moderate, and 17.37% felt dissatisfied or very dissatisfied with current job. Medical staff was evidently less satisfied with compensation packages (18.55% only) and learning and training opportunities (18.29% only) than other work-related aspects (Table 3).

**Table 3:** **Assessment on the factors related to job satisfaction of medical staff in pilot county hospitals**

**Sociodemographic characteristics, working situation, and satisfaction on work-related factors**	**Job satisfaction of medical staff in pilot county hospitals (** ***n*** **= 1531)**										***χ*** ^**2**^	***P***
	**Very satisfied**		**Satisfied**		**Moderate**		**Dissatisfied**		**Very dissatisfied**			
	***n***	**%**	***n***	**%**	***n***	**%**	***n***	**%**	***n***	**%**		
Gender												
Male	44	7.97	210	38.04	196	35.51	88	15.94	14	2.54		
Female	92	9.40	354	36.16	369	37.69	151	15.42	13	1.33	4.588	0.332
Age												
24 and below	14	9.33	48	32.00	65	43.33	21	14.00	2	1.33		
25–34	45	8.54	169	32.07	216	40.99	84	15.94	13	2.47		
35–44	40	7.16	211	37.75	207	37.03	93	16.64	8	1.43		
45–54	30	11.19	123	45.90	75	27.99	37	13.81	3	1.12		
55 and above	7	25.93	13	48.15	2	7.41	4	14.81	1	3.70	44.291	<0.001
Educational background												
Technical secondary school and below	32	17.02	64	34.04	61	32.45	28	14.89	3	1.60		
Junior college	49	8.96	210	38.39	209	38.21	75	13.71	4	0.73		
Bachelor’s degree	54	7.33	278	37.72	265	35.96	122	16.55	18	2.44		
Master’s degree or above	1	1.69	12	20.34	30	50.85	14	23.73	2	3.39	39.668	<0.001
Position												
Doctor	40	6.68	208	34.72	215	35.89	116	19.37	20	3.34		
Nurse	58	8.77	242	36.61	257	38.88	98	14.83	6	0.91		
Medical technician	38	14.02	114	42.07	93	34.32	25	9.23	1	0.37	42.361	<0.001
Professional title												
No title	3	12.50	7	29.17	12	50.00	2	8.33	0	0.00		
Junior title	40	7.80	159	30.99	216	42.11	86	16.76	12	2.34		
Middle title	68	9.33	288	39.51	256	35.12	107	14.68	10	1.37		
Senior title	25	9.43	110	41.51	81	30.57	44	16.60	5	1.89	21.666	0.041
Years in professional working experience												
1–5 years	26	9.03	81	28.13	123	42.71	52	18.06	6	2.08		
6–10 years	17	7.94	64	29.91	91	42.52	35	16.36	7	3.27		
11–15 years	13	5.73	93	40.97	89	39.21	30	13.22	2	0.88		
16–20 years	27	8.79	111	36.16	113	36.81	50	16.29	6	1.95		
20 years and above	53	10.71	215	43.43	149	30.10	72	14.55	6	1.21	38.586	0.001
City												
Wuhan city	17	7.73	87	39.55	81	36.82	33	15.00	2	0.91		
Jingzhou city	12	5.50	82	37.61	84	38.53	37	16.97	3	1.38		
Yichang city	20	9.30	78	36.28	73	33.95	40	18.60	4	1.86		
Xiangyang city	22	10.00	72	32.73	89	40.45	29	13.18	8	3.64		
Shiyan city	23	10.50	77	35.16	78	35.62	34	15.53	7	3.20		
Suizhou city	16	7.31	78	35.62	86	39.27	38	17.35	1	0.46		
Jingmen city	26	11.82	90	40.91	74	33.64	28	12.73	2	0.91	27.146	0.298
The number of hours spent at work everyday												
8 h and below	97	13.16	308	41.79	260	35.28	66	8.96	6	0.81		
9 h	26	6.39	148	36.36	155	38.08	74	18.18	4	0.98		
10 h	9	4.11	65	29.68	84	38.36	56	25.57	5	2.28		
11 h	1	2.17	13	28.26	23	50.00	7	15.22	2	4.35		
12 h	1	2.70	8	21.62	17	45.95	9	24.32	2	5.41		
12 h and above	2	2.35	22	25.88	26	30.59	27	31.76	8	9.41	141.127	<0.001
The degree of work stress												
No pressure	14	45.16	10	32.26	5	16.13	2	6.45	0	0.00		
Slight pressure	56	13.63	194	47.20	129	31.39	27	6.57	5	1.22		
Moderate pressure	29	12.03	91	37.76	96	39.83	25	10.37	0	0.00		
Considerable pressure	34	4.99	225	32.99	287	42.08	126	18.48	10	1.47		
Extreme pressure	3	1.81	44	26.51	48	28.92	59	35.54	12	7.23	222.417	<0.001
Satisfaction on performance appraisal system												
Very satisfied	22	64.71	4	11.76	6	17.65	1	2.94	1	2.94		
Satisfied	80	16.84	257	54.11	107	22.53	30	6.32	1	0.21		
Moderate	28	3.57	258	32.91	364	46.43	126	16.07	8	1.02		
Dissatisfied	5	2.54	42	21.32	74	37.56	67	34.01	9	4.57		
Very dissatisfied	1	2.44	3	7.32	14	34.15	15	36.59	8	19.51	483.816	<0.001
Satisfaction on concern showed by leaders												
Very satisfied	35	46.05	30	39.47	9	11.84	2	2.63	0	0.00		
Satisfied	79	15.28	258	49.90	132	25.53	47	9.09	1	0.19		
Moderate	19	2.45	132	17.03	363	46.84	135	17.42	12	1.55		
Dissatisfied	2	1.50	47	35.34	56	42.11	42	31.58	8	6.02		
Very dissatisfied	1	3.33	1	3.33	5	16.67	13	43.33	6	20.00	427.387	<0.001
Satisfaction on hospital management												
Very satisfied	38	49.35	30	38.96	9	11.69	0	0.00	0	0.00		
Satisfied	79	12.99	311	51.15	165	27.14	50	8.22	3	0.49		
Moderate	17	2.28	204	27.35	364	48.79	148	19.84	13	1.74		
Dissatisfied	1	1.20	18	21.69	25	30.12	31	37.35	8	9.64		
Very dissatisfied	1	5.88	1	5.88	2	11.76	10	58.82	3	17.65	466.78	<0.001
Satisfaction on compensation packages												
Very satisfied	11	55.00	7	35.00	1	5.00	0	0.00	1	5.00		
Satisfied	60	22.73	139	52.65	57	21.59	8	3.03	0	0.00		
Moderate	39	5.53	286	40.57	294	41.70	81	11.49	5	0.71		
Dissatisfied	24	5.22	123	26.74	186	40.43	116	25.22	11	2.39		
Very dissatisfied	2	2.44	9	10.98	27	32.93	34	41.46	10	12.20	363.14	<0.001
Satisfaction on learning and training opportunities												
Very satisfied	11	32.35	11	32.35	10	29.41	1	2.94	1	2.94		
Satisfied	42	17.07	114	46.34	69	28.05	20	8.13	1	0.41		
Moderate	48	9.14	218	41.52	178	33.90	76	14.48	5	0.95		
Dissatisfied	22	4.57	147	30.56	204	42.41	94	19.54	14	2.91		
Very dissatisfied	13	5.31	74	30.20	104	42.45	48	19.59	6	2.45	112.753	<0.001

Through Pearson chi-square statistical analysis, the results showed that factors related to the job satisfaction of medical staff in pilot county hospitals included age, educational background, position, professional title, years in professional working experience, the number of hours spent at work, work stress, and satisfaction on the following aspects: performance appraisal system, concern showed by leaders, hospital management, compensation packages, and learning and training opportunities (*P* < 0.05; Table 3).

To further determine the significant factors influencing the job satisfaction of medical staff in pilot county hospitals, we substituted the variables with statistical significance from the previous Pearson chi-square statistical analysis into the binary logistic regression model for calculation. The dependent variable *Y* in logistic regression included the following: 1 “satisfied” (very satisfied, satisfied, moderate) and 0 “dissatisfied” (dissatisfied, very dissatisfied). The independent variable *X*1 (age) included five grades from younger to older. The independent variable *X*2 (position) included “doctor”, “nurse”, and “medical technician”. The independent variable *X*3 (professional title) included four grades from lower to higher. The independent variable *X*4 (years in professional working experience) included five grades from shorter to longer. The independent variable *X*5 (educational background) included four grades from lower to higher. The independent variable *X*6 (the number of hours spent at work every day) included four grades from less to greater. The independent variable *X*7 (work stress) included two grades: 1 “no pressure or moderate pressure” (no pressure, slight pressure, moderate pressure) and 2 “great pressure” (considerable pressure, extreme pressure). The independent variables *X*8 (satisfaction on performance appraisal system), *X*9 (satisfaction on concern showed by leaders), *X*10 (satisfaction on hospital management), *X*11 (satisfaction on compensation packages), and *X*12 (satisfaction on learning and training opportunities) all included two grades: 1 “satisfied” (very satisfied, satisfied, moderate) and 2 “dissatisfied” (dissatisfied, very dissatisfied).

The results of the binary logistic regression analysis showed that the factors significantly influencing the job satisfaction of medical staff in pilot county hospitals included the number of hours spent at work, work stress, satisfaction on performance appraisal system, satisfaction on hospital management, satisfaction on compensation packages, and satisfaction on learning and training opportunities (*P* < 0.05; Table 4). In particular, the job satisfaction probability of the medical staff who worked for 9, 10, and 11 h every day were 0.483 times (OR = 0.483, 95% CI = 0.327–0.713, *P* < 0.001), 0.364 times (OR = 0.364, 95% CI = 0.230–0.576, *P* < 0.001), and 0.339 times (OR = 0.339, 95% CI = 0.202–0.568, *P* < 0.001) lower than that of the medical staff who worked for a maximum of 8 h, respectively. The job satisfaction probability of the medical staff who felt no pressure or moderate pressure was 2.384 times higher (OR = 2.384, 95% CI = 1.666–3.411, *P* < 0.001) than that of the medical staff who felt great work pressure. These results showed that working hours and work pressure were negatively related to job satisfaction in pilot county hospitals. The job satisfaction probability of the medical staff who were satisfied with the performance appraisal system was 2.243 times higher than that of the medical staff who were dissatisfied (OR = 2.243, 95% CI = 1.504–3.345, *P* < 0.001). The job satisfaction probability of the medical staff who were satisfied with hospital management was 2.043 times higher than that of the medical staff who were dissatisfied (OR = 2.043, 95% CI = 1.196–3.490, *P* = 0.009). The job satisfaction probability of the medical staff who were satisfied with compensation packages was 3.298 times higher than that of the medical staff who were dissatisfied (OR = 3.298, 95% CI = 2.410–4.513, *P* < 0.001). The job satisfaction probability of the medical staff who were satisfied with learning and training opportunities was 1.442 times higher than that of the medical staff who were dissatisfied (OR = 1.442, 95% CI = 1.050–1.980, *P* = 0.024). These results showed that satisfaction on performance appraisal system, hospital management, compensation packages, and learning and training opportunities were positively related to job satisfaction in county hospitals.

**Table 4:** **Analysis on the multiple factors influencing job satisfaction of medical staff in pilot county hospitals**

**Medical staff in pilot county hospitals**	**Reference category**	***B***	***P***	**OR**	**95% CI**
Age	24 and below				
25–34		−0.231	0.522	0.794	(0.392–1.610)
35–44		−0.549	0.279	0.577	(0.213–1.562)
45–54		−0.603	0.309	0.547	(0.171–1.748)
55 and above		−0.91	0.279	0.403	(0.077–2.093)
Position	Medical technician				
Doctor		−0.368	0.201	0.692	(0.394–1.216)
Nurse		−0.39	0.139	0.677	(0.404–1.136)
Professional title	No title				
Junior title		−0.249	0.719	0.779	(0.201–3.027)
Middle title		−0.458	0.506	0.633	(0.164–2.437)
Senior title		−0.417	0.555	0.659	(0.165–2.635)
Years in professional working experience	1–5 years				
6–10 years		0.36	0.242	1.434	(0.784–2.623)
11–15 years		0.72	0.054	2.055	(0.989–4.272)
16–20 years		0.601	0.200	1.823	(0.728–4.567)
20 years and above		0.559	0.280	1.748	(0.635–4.817)
Educational background	Technical secondary school and below				
Junior college		0.344	0.228	1.411	(0.806–2.469)
Bachelor’s degree		0.35	0.261	1.42	(0.771–2.615)
Master’s degree or above		0.352	0.464	1.421	(0.554–3.644)
The number of hours spent at work everyday	8 h and below				
9 h		−0.727	<0.001	0.483	(0.327–0.713)
10 h		−1.01	<0.001	0.364	(0.230–0.576)
11 h and above		−1.083	<0.001	0.339	(0.202–0.568)
The degree of work stress	Great pressure				
No pressure or moderate pressure		0.869	<0.001	2.384	(1.666–3.411)
Satisfaction on performance appraisal system	Dissatisfied				
Satisfied		0.808	<0.001	2.243	(1.504–3.345)
Satisfaction on concern showed by leaders	Dissatisfied				
Satisfied		0.302	0.206	1.352	(0.848–2.157)
Satisfaction on hospital management	Dissatisfied				
Satisfied		0.715	0.009	2.043	(1.196–3.490)
Satisfaction on compensation packages	Dissatisfied				
Satisfied		1.193	<0.001	3.298	(2.410–4.513)
Satisfaction on learning and training opportunities	Dissatisfied				
Satisfied		0.366	0.024	1.442	(1.050–1.980)

### Understanding and perception of the public hospital reform of medical staff in pilot and non-pilot county hospitals

In pilot county hospitals, only 35.92% of the investigated medical staff members expressed that they knew a lot or fully knew of the specific contents of the public hospital reform programme and policy. Only 50.29% of the investigated medical staff members expressed that they obviously perceived of the promotion of public hospital reform in their county. Approximately 59.24% of the investigated medical staff members expressed that obvious changes occurred in their hospitals since the reform began in 2008. Only 8.10% of the investigated medical staff members considered that the reform could significantly solve the main problems in public hospitals, and 68.32% considered that the reform could solve only part of the problems. Only 6.27% of the investigated medical staff thought that the reform could have evident effects that could solve the difficulty in the accessibility of medical service, and 71.52% thought that this reform could affect and alleviate this difficulty only to some extent. Only 7.25% of the investigated medical staff thought that the reform could have evident effects that could solve the difficulty in the affordability of medical service, and 66.75% thought that this reform could only alleviate this difficulty to some extent.

The Pearson chi-square test method was used to assess the differences in the understanding and perception of the reform between medical staff in pilot county hospitals and medical staff in non-pilot county hospitals. The results showed that medical staff in pilot and non-pilot county hospitals exhibited significant differences in all the aspects aforementioned (*P* < 0.05; Table 5)

**Table 5:** **The understanding and perception of public hospital reform of medical staff in pilot and non-pilot county hospitals**

**The understanding and perception of public hospital reform**	**Pilot county hospitals (** ***n*** **= 1531)**		**Non-pilot county hospitals (** ***n*** **= 737)**		**Total**		***χ*** ^***2***^	***P***
	***n***	**%**	***n***	**%**	***n***	**%**		
The understanding degree of specific contents of the public hospital reform programme								
Fully know	70	4.57	14	1.90	84	3.70		
Know a lot	480	31.35	177	24.02	657	28.97		
Know some	484	31.61	304	41.25	788	34.74		
Know a little	424	27.69	201	27.27	625	27.56		
Know nothing	73	4.77	41	5.56	114	5.03	32.787	<0.001
The perceived level of the reform promotion in this county								
Perceive obviously	770	50.29	293	39.76	1063	46.87		
Perceive not obviously	578	37.75	370	50.20	948	41.80		
Perceive nothing	46	3.00	22	2.99	68	3.00		
Have no idea	137	8.95	52	7.06	189	8.33	32.378	<0.001
The perceived level of changes brought by reform since 2008								
Change obviously	907	59.24	231	31.34	1138	50.18		
Change not obviously	494	32.27	374	50.75	868	38.27		
Change nothing	65	4.25	100	13.57	165	7.28		
Have no idea	65	4.25	32	4.34	97	4.28	181.017	<0.001
If public hospital reform could solve the problems faced by public hospitals								
Solve the main problems	124	8.10	38	5.16	162	7.14		
Solve only part of the problems	1046	68.32	491	66.62	1537	67.77		
Solve nothing	136	8.88	94	12.75	230	10.14		
Have no idea	225	14.70	114	15.47	339	14.95	13.797	0.003
If public hospital reform could solve the difficulty in accessibility of medical service								
Have obvious effects	96	6.27	27	3.66	123	5.42		
Have some effects	1095	71.52	469	63.64	1564	68.96		
Have no effect	175	11.43	132	17.91	307	13.54		
Have no idea	165	10.78	109	14.79	274	12.08	32.783	<0.001
If public hospital reform could solve the difficulty in affordability of medical service								
Have obvious effects	111	7.25	24	3.26	135	5.95		
Have some effects	1022	66.75	428	58.07	1450	63.93		
Have no effect	226	14.76	175	23.74	401	17.68		
Have no idea	172	11.23	110	14.93	282	12.43	47.353	<0.001

## Discussion

### Medical staff members in pilot county hospitals were exposed to work-related stress to a higher extent

In this study, medical staff members in pilot county hospitals were exposed to work-related stress at a higher extent than those in non-pilot county hospitals. This result could be attributed to the increasing number of patients, higher requirements for technical level and comprehensive quality of medical staff, and reform measures of hospital administration system caused by the public hospital reform [6]. Because of the health reform, the basic social health insurance system has been improved and medical cost and expenses have been reasonably controlled. The medical demand of the population is released and medical staff members would see more patients/day. In China, many jobs require employees to work for only 8 h a day, but the results of this study showed that more than half of the medical staff members (51.86%) work for more than 8 h a day in pilot county hospitals. This finding showed that overtime working is common for medical staff in pilot county hospitals, which may increase the work stress.

### Pilot county hospitals have implemented some reform measures and got some positive effects, but there still are deficiencies

Medical staff members in pilot county hospitals were more satisfied on current job, performance appraisal system, concern showed by leaders, hospital management, and compensation packages than those in non-pilot county hospitals. This finding suggested that the pilot county hospitals have implemented some reform measures to improve performance appraisal system and management system, to concern more on their staff, and to increase income and job satisfaction with this reform. These measures have showed some positive effects. However, there was no significant difference between satisfaction on learning and training opportunities of medical staff in pilot county hospitals than that in non-pilot county hospitals. This suggested that pilot county hospitals have not implemented sufficient effective measures to provide enough learning and training opportunities to medical staff.

### Medical staff members in pilot county hospitals have evidently less satisfaction on compensation packages and learning and training opportunities

Within pilot county hospitals, medical staff members were evidently less satisfied with compensation packages and learning and training opportunities than with other work-related aspects. These indicated that medical staff members considered that current remuneration did not match the amount of work, and the learning and training opportunities currently provided for them did not satisfy the demands. At present, the average annual income of medical staff in county hospitals in China is basically the same as the average annual income of urban workers. But in many other counties, the income of medical staff is generally higher than that of other professions and is four or five times the income of urban workers. The income level of medical staff in county hospitals in China is well below the international situation. Hence, medical staff urgently needs improvement. In China, the income levels of medical staff in different county hospitals are basically the same. Therefore, dissatisfaction on current remuneration of medical staff in county hospital is a common problem.

### Significant influencing factors of job satisfaction of medical staff in pilot county hospitals have been determined

Job satisfaction originates in the organizational psychology literature but has been adopted by some researchers in the field of human resources for health [7-9]. Locke defined this concept as “a pleasurable or positive emotional state resulting from the appraisal of one’s job or job experiences” [10]. In the field of health care, job satisfaction of medical staff determines the quality of service delivery to patients [11,12]. Poor job satisfaction is associated with absenteeism, employee turnover in an organization, and eventual exhaustion [13,14].

To assess job satisfaction, we further analysed the factors influencing the job satisfaction of medical staff in pilot county hospitals by univariate and multivariate analyses. Univariate analysis results showed that sociodemographic characteristics (including age, educational background, position, professional title, and years in professional working experience), working situation (including the number of hours spent at work every day and work stress), and satisfaction on some work-related factors (including satisfaction on performance appraisal system, concern showed by leaders, hospital management, compensation packages, and learning and training opportunities) were related to the job satisfaction of medical staff in pilot county hospitals.

However, multivariate analysis results showed that only the number of hours spent at work, work stress, and satisfaction on performance appraisal system, hospital management, compensation packages, and learning and training opportunities were significantly associated with job satisfaction of medical staff in pilot county hospitals. None of the sociodemographic characteristics exhibited a significant correlation. It indicated that the influence on job satisfaction of medical staff caused by sociodemographic characteristics in this study was weaker than that caused by working situation and satisfaction on work-related factors. This phenomenon could be attributed to great changes in the working situations of medical staff and work-related factors in pilot county hospitals in China as a result of the implementation of the health-care reform and hospital reform. Therefore, sociodemographic characteristics did not strongly influence job satisfaction, whereas working situations and satisfaction on work-related factors were statistically significant.

The results of multivariate analysis indicated long hours spent at work each day and high work-related stress encountered could result in less job satisfaction. By comparison, a high satisfaction on performance appraisal system, hospital management, compensation packages, and learning and training opportunities could indicate high job satisfaction. These findings are consistent with those in earlier studies on job satisfaction [14-28]. For example, health workers in Ghana overwhelmingly identify low salaries as the main source of dissatisfaction on an interviewer-administered questionnaire [29]. Kumar and co-workers found that factors influencing the satisfaction level include low salaries, lack of training opportunities, improper supervision, and inadequate financial rewards. Marinucci and co-workers conducted a survey in seven sub-Saharan African countries [30], and the result shows that professional development and training opportunities are the most important factor resulting in job satisfaction as indicated by approximately 90% of the total interviewees. Peters and his co-workers conducted a survey in India and found that many employees rate “training opportunities” as one of the motivating factors [31]. A review of 12 empirical studies on the motivation of developing and developed countries has found that seven major job characteristics are important determinants of job motivation, including opportunities for personal development, pay/rewards, management practices, and organizational policies [32]. A study in Vietnam has also found that the main motivating factors of health workers include the following: the appreciation expressed by their managers, competitive income, and training [33].

### Suggestions for improving job satisfaction of medical staff in pilot county hospitals

Therefore, the government officials and hospital administrators should pay attention to these influencing factors and focus on the demands of medical staff in pilot county hospitals. What should be done most at present mainly include three aspects [34,35]. 1) The hospital administrators should have more concern for the working situation of medical staff in pilot county hospitals, properly assign and arrange work, and appropriately reduce workload and work stress to promote job satisfaction and active participation of medical staff in pilot county hospitals. 2) The government officials and hospital administrators should improve the system of compensation packages, promote income levels, and make the income match the workload and technical value of medical staff in pilot county hospitals. 3) More learning and training opportunities should be provided and created to medical staff in pilot county hospitals, in order to help them to improve their professional level and meet their individual development requirements. 4) More physical and mental health considerations and better performance appraisal system and management system should be provided for medical staff in pilot county hospitals, in order to promote their job satisfaction. 5) In a previous study, the participation of medical staff in decision-making significantly affected job satisfaction [28]; as such, democratic management can be applied in pilot county hospitals.

### Medical staff in pilot county hospitals exhibited better understanding of the public hospital reform programme and more positive attitude towards it, but it still needs improvement

The results of the data analysis showed that the medical staff in pilot county hospitals exhibited a better understanding of the specific contents of the public hospital reform programme, more optimistic perception of the changes caused by the reform, and more firm confidence and positive attitude towards the reform compared with the medical staff in non-pilot county hospitals. These findings indicated that the reform measures implemented in pilot county hospitals have resulted in some positive effect, and the medical staff in pilot county hospitals experienced more advantages from this reform. However, there still were some medical staff members in pilot county hospitals who showed insufficient understanding and perception of the reform and hold negative attitude towards the effect of the reform. These findings suggested that the understanding, perception, and attitude towards public hospital reform of the medical staff in pilot county hospitals still need improvement. Therefore, 1) the government should provide more implementing rules of the reform policy, to make the reform policy more clearly and operable; 2) the government officials and hospital administrators can guide medical staff members with different individual characters by using different methods to learn the knowledge and importance of this reform in pilot county hospitals; 3) to promote the active implementation of public hospital reform, government officials and hospital administrators should guide and encourage the medical staff in pilot county hospitals to take part in the reform, and make more publicity on the benefits of the reform utilizing multiple forms, such as conferences, posters, and TV shows.

### Possible limitations

In this study, there were three possible limitations: first, the cross-sectional design with job satisfaction. It was difficult to establish a causal conclusion, and the longitudinal survey might be carried out to confirm the causal conclusion in our future study. Second, the measurements were performed by a self-administrated method. Then, it is possible that the respondents might have overreported or underreported their level of job satisfaction and satisfaction on work-related factors and understanding and perception of the reform. Third, given that the study was conducted only in counties of Hubei province, the findings of the study may or may not be generalized to medical staff working in other areas in China.

## Conclusions

The results in this study indicated that pilot county hospitals have implemented some measures to improve the performance appraisal system and management system, provide adequate care for their medical staff, and increase medical staff’s income and job satisfaction through the reform. Pilot county hospitals have experienced some positive effects but there still are deficiencies. Within pilot county hospitals, work stress increased and less than half of the medical staff members were very satisfied or satisfied with current job. To promote the job satisfaction of medical staff in pilot county hospitals, the government officials and hospital administrators should pay attention to these influencing factors of job satisfaction and focus on the reasonable demands of medical staff in pilot county hospitals.

In addition, the medical staff members in pilot county hospitals exhibited a better understanding of the public hospital reform programme and showed more firm confidence and positive attitude towards the reform than the medical staff members in non-pilot county hospitals. These findings indicated that the reform measures implemented in pilot county hospitals have resulted in some positive effect, and the medical staff in pilot county hospitals experienced more advantages from this reform. However, there still were some medical staff members in pilot county hospitals who showed insufficient understanding and perception of the reform and hold negative attitude towards the effect of the reform. These findings suggested that the understanding, perception, and attitude towards the public hospital reform of the medical staff and the publicity and education of the reform in pilot county hospitals still need improvement.
